# Enactivism and Ecological Psychology: The Role of Bodily Experience in Agency

**DOI:** 10.3389/fpsyg.2020.539841

**Published:** 2020-10-15

**Authors:** Yanna B. Popova, Joanna Rączaszek-Leonardi

**Affiliations:** ^1^Polish Institute of Advanced Studies (PIASt), Warsaw, Warsaw, Poland; ^2^Human Interactivity and Language Lab, Faculty of Psychology, University of Warsaw, Poland

**Keywords:** ecological psychology, enactivism, embodiment, experience, social cognition, art reception, kinaesthesia, agency

## Abstract

This paper considers some foundational concepts in ecological psychology and in enactivism, and traces their developments from their historical roots to current preoccupations. Important differences stem, we claim, from dissimilarities in how embodied experience has been understood by the ancestors, founders and followers of ecological psychology and enactivism, respectively. Rather than pointing to differences in domains of interest for the respective approaches, and restating possible divisions of labor between them in research in the cognitive and psychological sciences, we call for a deeper analysis of the role of embodiment in agency that we also undertake. Awareness of the differences that exist in the respective frameworks and their consequences, we argue, may lead to overcoming some current divisions of responsibility, and contribute to a more comprehensive and complementary way of dealing with a broader range of theoretical and practical concerns. While providing some examples of domains, such as social cognition and art reception, in which we can observe the relative usefulness and potential integration of the theoretical and methodological resources from the two approaches, we demonstrate that such deeper synergy is not only possible but also beginning to emerge. Such complementarity, as we envisage, conceives of ecological psychology that allows felt experience as a crucial dynamical element in the explanations and models that it produces, and of an enactive approach that takes into consideration the ubiquitous presence of rich directly perceived relations among variables arising from enactments in the social and physical world.

## Introduction

This article aims to consider some foundational concepts in enactivism and in ecological psychology, and how they have come to shape the past and current concerns of the main theorists in these respective frameworks. Both theories are currently described as positions of “radical embodiment” (Baggs and Chemero, 2018) in that they both reject a representational view of human cognitive processes and see being and acting in the world as a foundational capability of the mind. This suggests, we think, more than simply an accidental convergence of ideas, and indicates a common historical ancestry of a kind, one which remains largely unexplored, namely, the radical empiricism of William James on the one hand and a broadly phenomenological understanding of the activity of the body as foundational for perception and cognition on the other. As some researchers have already discussed (Glotzbach and Heft, 1982; Heft, 1989; Chemero and Käufer, 2016), despite coming from different traditions, Gibson was familiar with the work of Merleau-Ponty and used some related ideas in his own research. Radical empiricism, as developed by William James, was also a significant influence on Gibson, and the common threads between James and key phenomenologists still remain a point of intense historical and theoretical debate. Enactivism has openly and repeatedly acknowledged its links with phenomenology and thinkers such as Husserl, Jonas, and Merleau-Ponty. Nevertheless, our aim here is not merely looking for direct influences but, rather, highlighting a certain creative confluence and a commonality of ideas that shaped and continue to inform these two influential positions in current research in psychology, philosophy, and cognitive science. We propose that in order to examine the similarities and differences between the two approaches and seek the points of possible mutual support, a deeper analysis is needed of the crucial notion of experience, which, in turn, determines how embodiment and agency are understood. This is driven in part by the fact that a lot of current work that ventures outside of narrowly understood cognition into areas such as architecture, art and art reception, or media and narrative in general tends to use enactivist approaches and Gibson’s theory of affordance interchangeably, without much precision. A similar situation exists in relation to discussions about social cognition, where the process of perceiving “social affordances” and the enactivist concept of “participatory sense-making” appear to be used as analogous. It is our aim in this article to show how through careful considerations of the notions of embodied experience and agency certain choices of applications to diverse fields such as social cognition, art reception, or psychopathology, to name but a few, can become better understood and perhaps more thoroughly evaluated and differentiated. The complementarity of the approaches that is increasingly often being pointed to and/or called for (Baggs and Chemero, 2018; Heras-Escribano, 2019) has, we claim, to rest on a clarified common conceptual core in order to have the potential of becoming a strong alternative to representational approaches. At present, within cognitive and psychological sciences, the applicability of ecological psychology seems to be more in the broadly understood action-perception and motor-coordination field, providing multiple methodologies and tools for empirical research, while enactivist approaches seem to be especially helpful in the domain of social interaction, including psychopathology and art reception, as well as in setting up the philosophical frame for researchers. Perhaps a more nuanced understanding of the relation between experience, embodiment, and agency would lead to pooling of the respective expertise and methods so that they can be applied in a broader range of common domains. In the second part of the article we attempt to show such advantages on the example of early social interaction and art reception.

## Sources and Developments

It is important to address briefly the issue of how particular ideas developed in reaction to accepted views at the time of conception in both the early enactivism of Varela et al. (1991) and in Gibson’s ecological theory of perception (1966, 1979), and how these positions of carving a particular intellectual response to dominant views gave rise to subsequent emphases on specific aspects that led to divergences as well as commonalities in the two theoretical approaches under consideration.

### Enactivism

As acknowledged by Varela et al. (1991) and Thompson (2007), early enactivism was mainly formulated as a reaction against classical, first-generation Cognitivism, which was based on the prevalent-at-the-time computational theory of mind (Thompson, 2007, pp. 4–8). Early enactivism therefore was explicitly framed as a rejection of information processing and symbolic representations that dominated cognitive science, and sought ways to reconcile the scientific study of the mind with the lived experience of an organism. One main criticism was aimed at the cognitivist failure to account for such subjective experience, providing instead a model of the brain as a “stimulus-driven, sequential processing computer” (Thompson, 2016, p. xix). Another major point of contention was the evident lack of interest, shown by cognitivists, in the role of not just bodily but also environmental and social dynamics in cognition and lived experience. To that extent, enactivism came as a position of radical change in our understanding of mind and life in cognitive science, and as such it reached to phenomenology in order to emphasize the role of embodiment, embeddedness, and enactment for understanding cognitive processes. As Evan Thompson acknowledges, many things have changed since those early days of enactivism. Embodiment has become a central concern for cognitive science, as has the self-organizing and distributed nature of brain processes, and “the deep continuity in the principles of self-organization from the simplest living things to more complex cognitive beings” (Thompson, 2016, p. xix).

For our purposes here it is crucial to highlight the lessons from phenomenology that have shaped the past and continue to contribute to current work within enactivism. Phenomenology studies pre-reflective as well as conscious lived experiences, and respects the centrality of the first-person point of view. It is best understood as a strictly defined commitment to the role played by subjectivity in the constitution of everything that we do, including scientific projects (see Moran, 2000; Sokolowsky, 2000). The core term of phenomenology is “intentionality,” understood as the claim that all acts of consciousness, be it perceptions, feelings, moods, decisions, memories, or imaginations, are experiences of something. Our awareness is inescapably linked with the world of things and other people. Phenomenological description is therefore always an intentional description, revealing the inherent relationship of the world with subjectivity^1^. This is precisely how the initial formulation of enactivism as “the emergence of mind as entailing the emergence of a world” can be properly understood. It can be argued that because phenomenology is primarily understood as a philosophy of experience, its influence filters directly into preoccupations in early and contemporary enactivism with issues such as individuation, autonomy, and agency, as we will discuss below. For example, the five principles, highlighted by Thompson (2007, p. 13) as the main ideas behind the theory, show explicitly that enactivism foregrounds the constitutive nature of subjective experience in relation to an external realm that is “brought forth by a living being’s autonomous agency.” Importantly, subjective experience is not seen as “an epiphenomenal side issue, but central to the understanding of the mind” (Thompson, 2007, p. 13). It needs to be emphasized, however, that with its commitment to the biological study of the self-organization of all living beings, enactivism goes beyond the object matter of phenomenology in its preoccupation with the constitutive nature of subjective experience, as seen in more recent work in enactivism (e.g., Di Paolo et al., 2017). This is to say that the self-organizational nature of biological structure and the foundational role of embodied subjectivity are equally important tenets of enactivism (see also Stewart et al., 2010)^2^.

So, how are the lessons from phenomenology manifested in enactivism? What are the contributions of a phenomenological description of subjective experience to enactivism as a cognitive science discipline? Husserl understands *experience* or *lived experience* (a translation of the German verb *Erleben* and noun *Erlebnis*) as “something that one lives through,” “the conscious state as personally lived through and experienced in the first person” (Moran and Cohen, 2012, pp. 115, 195). It includes perception, imagination, memory, emotion, and many other aspects of conscious life. Each intentional act, such as judging, perceiving, wishing, and so on, has a particular quality to it, which is given in experience. Husserl also distinguishes between *lived experience* and the properties of the *mind-transcendent object* (Moran and Cohen, p. 169). This is because in lived experience we can only know the particular object in an incomplete way, as it presents itself to us in a certain aspect, from a particular angle, etc. However, it is the experience of an object that fundamentally underlies our encounter with it. As a well-known commentator on Husserl has described it, “[w]e are never conscious of an object *simpliciter*, but always of the object as appearing in a certain way; as judged, seen, described, feared, remembered, smelled, anticipated, tasted, and so on. We cannot be conscious of an object (a tasted lemon, a smelled rose, a seen table, a touched piece of silk) unless we are aware of the experience through which this object is made to appear (the tasting, smelling, seeing, touching)” (Zahavi, 2005, p. 121).

One fundamental aspect of lived experience is “embodiment,” understood most basically as the view that our knowledge of the world is inseparable from the experiences of the bodies that we are^3^. Preoccupation with the body is a major characteristic of phenomenology, but it is important to stress that the body for phenomenologists, such as Husserl and Merleau-Ponty, is not the body seen as any other object in the world, but precisely the felt and animated body experienced by a particular first-person perspective, in other words, the body-as-experiencer, the body-as-lived (*Leib*), and in contrast to the physical body (*Körper*) (Husserl, 1989). The lived body’s main characteristic is that it is always given as “my own body” in such a way that “I experience myself as ‘holding sway’ over this body” (Moran and Cohen, p. 194). The lived body, then, is not just a center of experience but a center of agency, or “willful self-movement.” As Husserl has described it, “the living body is never absent from the perceptual field” (quoted in Moran and Cohen, p. 194). This is clearly mirrored by Merleau-Ponty when he says: “[I]t is, therefore, quite true that any perception of a thing, a shape or a size as real, any perceptual constancy refers back to the positing of a world and of a system of experience in which my body is inescapably linked with phenomena. But the system of experience is not arrayed before me as if I were God, it is lived by me from a certain point of view; I am not the spectator, I am involved and it is my involvement in a point of view which makes possible both the finiteness of my perception and its opening out upon the complete world as a horizon for every perception” (Merleau-Ponty, 2002, pp. 353–354).

What enactivism takes from phenomenology (particularly from the philosophy of Husserl, Jonas, Merleau-Ponty) can be summed up by the statement that human experience is inherently incarnate (embodied) and the study of embodiment revolves around questions of action, perception, and motility. In current enactivist thinking cognition is thus defined as “the exercise of skillful know-how in situated and embodied action” (Thompson, 2007, p. 13). As discussed, both Husserl and Merleau-Ponty give special importance to the role of bodily motility in perception, and both emphasize the implicit, usually pre-reflectively functioning bodily intentionality that underlies everything that we do. The body is the sole vehicle for having a world, and this very aspect of incarnate existence is what is at the core of enactivist concern with experience. According to Thompson (2005, p. 11), any scientific account of the body as a locus of convergence of perception and action requires an account of not just selfhood or agency but also an account of a pre-reflectively known embodiment. In current enactivist work (e.g., Buhrmann and Di Paolo, 2015) this avowed preoccupation with embodiment has given rise to a complex understanding of what constitutes agency, which includes both a phenomenologically derived embodied selfhood and a biologically based view of the organism as a “self-organizing system.”

Within the perceptual world the body can appear as just another object to be perceived and examined, an “object body,” or *Körper*. Yet, inescapably, this is always accompanied by the experience of the body-as-lived, the body from within. For Merleau-Ponty human existence is thus “doubly” embodied, and this dual perspective between the physical body and the lived body provides a way to escape dualism in the description of embodied experience and even a way of reconciling a more scientific third-person stance and a first-person phenomenological one^4^. As first noticed by Husserl and later emphasized by Merleau-Ponty, it is intrinsic to lived embodiment to be both a subject of experience and an object available to one’s own as well as the other’s gaze. The body (*Leib*) thus performs a key role in the formation of intersubjectivity since the simultaneity of my experience of my body as both subject and object gives rise to the recognition of the subjectivity and lived embodiment of another. This feature of “embodiment” becomes particularly relevant in relation to “social affordances” and how an agent becomes, through her own spontaneous actions, a possibility for participatory action for others, as we will discuss below.

Another major theme from phenomenology that is evident in enactivism is the relational nature of the subject and world connection. Both embodiment and such relationality between organism and world are aspects of what constitutes experience, in other words, of how we perceive and act in the world, engage with other people, or, simply, live as an individual biological system. Embodiment, or having the body we do, determines not only what we perceive and how we act and think, but how the world appears or feels. With respect to relationality, as already mentioned in connection with intentionality, the mind and the world are not understood as two pre-given and discrete entities, but as “mutually constituted” in a dynamic and active relationship. Husserl first addressed the enworlded nature of experience, “the world as the ever present horizon of experiences” (Moran and Cohen, p. 189), and this theme was taken up by his followers in concepts such as “being-in-the-world” (Heidegger, 1962) and “being-to-the-world” (Merleau-Ponty, 2002). For both of these philosophers the subversion of classical dichotomies between subject and object, or subject and world, constitutes a substantial aspect of their respective projects in philosophy. For Heidegger, the very insertion of hyphens between the words of his key concept “being-in-the-world” (*In-der-Welt-sein*) serves as a graphic illustration of the conceptual disintegration of the dichotomy he seeks to overcome. In this mode of their thinking both philosophers can be seen as clear precursors of enaction. For example, E. Rosch’s own description captures this co-constitution of self and world, “[t]he environment of a given living body of whatever degree of complexity can only be what is knowable and known to its sense organs and cognitions, and that environment is in its turn constantly changed by the organism’s action on it – … neither side is pre-given” (Rosch, 2016, p. xxxviii). The same two themes of (i) embodiment and (ii) the relational nature of the subject/world connection are also dominant features of ecological psychology. Yet the role of felt experience in embodiment is different in ecological psychology, as is the emphasis on the direct perception of relations between subject and world and within the world itself, as we will show below.

### Ecological Psychology

Ecological psychology, an approach developed by J. J. Gibson, grew out of the radical empiricism of William James and the philosophical behaviorism of Edwin B. Holt. Although we recognize the complex, multithreaded nature of ecological psychology’s background (see Heft, 2001, for an excellent introduction to this background and history of the approach), including Gestalt psychology, and phenomenology, we focus on these roots as most closely linked to the issues we will pursue^5^. Following radical empiricism, it was a reaction against the mediated, inference-based theories of perception by a passive viewer (Heft and Richardson, 2013). While often thought to be mainly a theory of perception, ecological psychology, which followed from Gibson’s and his followers’ work, was based on redefining the organism/environment relationship, which meant a redefinition of most key concepts to cognition. As Heft points out, “there is hardly a topic in psychology for which considerations of the nature of the environment and an individual’s relation to it do not play an essential role” (Heft, 2001, p. 9). Thus, although Gibson’s and his followers’ interests are often linked to research on perception-action cycles in the domain of motor control, profound changes followed in thinking about such issues as the sources of meaningfulness of perception and action, the ways knowledge is gained and used, and the role of values in this process, thus making ecological psychology a comprehensive theoretical view and research framework (Gibson and Crooks, 1938; Gibson, 1966; Reed, 1988, 1996; Hodges, 2007). This broader engagement as a theory of cognition is evident in the scope of the theoretical debates that ecological psychologists initiated, for example, with the advocates of representational and modular versions of cognition (see, e.g., Fodor and Pylyshyn, 1981; Turvey and Carello, 1981; Turvey et al., 1981).

Ecological psychology is based on the assumption of an essentially active organism, where the coherence and adaptivity of action both shape and drive the cognitive processes. Perception is direct, continuous, and unmediated, and it involves the agent’s movement. The lack of the necessity for inferential processes stems from this directness and from the fact that perception provides rich and highly structured relational information, which is sufficient to specify behavior. The researcher’s task is to discover the properties of such relational dynamical structures and account for how they are meaningful for an agent, and how the agent’s actions are coupled to them, resulting in adaptive behavior.

The philosophical roots of Gibson’s theory lie in William James’s radical empiricism, which, as some theorists have claimed, served to pave the way also for phenomenology (Edie, 1970; Heft, 2001, p. 114). The core of radical empiricism is a refusal to acknowledge the distinctiveness and independence of organism and environment, knower and known, subject and object that we later find both in Husserl and in Merleau-Ponty. For James, the world possesses an inherent discoverable structure, which is directly apprehended and present in experience. The “radicality” of James’s empiricism stems from the proposal that what is present in the world and apprehended directly by experience is more than mere elements of the world, of which then the mind must make sense. Reality and the capacity to be experienced (experienceability) are granted also to relations and structures, which therefore do not have to be cognized in a separate cognitive feat: “Order is an intrinsic quality of encountering the world” (Heft, 2001, p. 36).

Although it is easy to see in these ideas the precursors for Gibson’s theory of direct perception, it is likely that influences from behaviorism made him emphasize only some of the consequences of such understanding of the relation between the world and an organism. In the ecological psychology framework, direct apprehension of the structures of the environment retains predominantly a functional value, serving to guide the organism’s actions^6^. The felt, subjective quality of the relational experience, it seems, has not been capitalized on or factored in the explanations of activities as an important feature. Thus, it can be safely stated that even though other influences on Gibson (such as Koffka or MacLeod) made him employ phenomenological description as a propaedeutic for experimental work, he was not a phenomenologist (Heft, 2001, pp. 114, 117).

The key concept in the ecological psychology view of cognition, which is presently proving itself increasingly useful both for the mainstream cognitivist approaches (see, e.g., Norman, 1999, for discussion) and certain newer enactivist/ecological hybrid approaches (e.g., Rietvelt and Kiverstein, 2014), is the notion of *affordances*. In an often quoted definition: “[t]he *affordances* of the environment are what it offers the animal, what it *provides* or *furnishes*, either for good or ill” (Gibson, 1979, p. 127). The role of the concept in the framework is to retain the action-relevance and organism’s subjectivity when talking about the perception of the world. Even though some understandings of affordances tend to objectivize them as features of the environment (Hutto and Myin, 2017), it seems rather clear that in most of Gibson’s writings they are a relational concept, in which both the environment and the organism are implicated (Chemero, 2003). Employing this concept realizes the tenets of James’s radical empiricism in a twofold way: first, affirming the subject/object inseparability, and second, granting reality and direct capacity to be perceived (experienceability) to relations. It is thus important to underscore the complexity of this notion. Affordances are relational in a double sense: (i) of unifying the organism and the environment, the knower and the known, but, also, (ii) of being based on complex, often non-obvious relations, which can be directly perceived. In later works in ecological psychology we witness further development and clarification of this unifying role of the concept of affordances.

One such important theoretical move, which served to make an explicit connection between ecological psychology and Merleau-Ponty’s phenomenological understanding on intentionality, was Heft’s (1989) consideration of the intentional nature of affordances and therefore of perception. It is misleading, he claimed, to treat affordances simply as causes for behavior. They should be thought of, rather, as constraints on actions that an animal is capable of producing, and is actually producing. Affordances thus scale not only to the action abilities or sizes of the animal’s bodies. Having a body is not having some average action vehicle but, as Heft reminds us, quoting from Merleau-Ponty: “having a body is […] to identify oneself with certain projects and to be continually committed to them” (Merleau-Ponty, p. 82, quoted in Heft, 1989). This allows us to understand affordances in relation to intentional acts. They are those environmental features that are implicated in ongoing projects, and it is from these intentional acts that they derive both their meaning and their capacity to be perceived. This seems to be a step toward explaining what it is to experience the sense of oneself as the author of one’s actions. This also alleviates the critique of the Gibsonian approach regarding culturally based affordances: adding to an intentional repertoire (e.g., via engagement in new routines, imitation, learning, or spontaneous behavior) brings about new affordances.

The embodied nature of the cognitive processes in ecological psychology thus stems from cognition understood as being for the action of a body and from taking activity as the starting point for cognition. It is the active body that shapes perceptual categories. The complexity of intentional acts in which an organism is involved can provide for the complex, relational, and multilayered nature of the perceivable and perceived properties of the world. However, it seems that Gibson’s approach focuses on merely a subset of projects and intentional acts in which we can be engaged, i.e., those connected to a goal-directed activity in the environment. The role of the body is thus mainly a functional role, and the embodiment of action does not refer to the felt experience this body possesses while acting. To be sure, the “I,” the self of an agent, is important: As described by Heft (2001, p. 120), “[a]ccompanying the experience of optical flow, and perception of the environment generally, is the experience that it is ‘I’ who is moving through the environment. This is not a Cartesian experience of the ‘I,’ a disembodied entity that is self aware as it thinks. This ‘I’ is much more concrete than that; it is the source of action and it can literally be seen by the perceiver. It is ‘I’ as purposive agent.” However, the indications of one’s bodily presence are not the felt bodily experiences but rather “persistent features in the field of view,” such as occluding edges. For example, the movements of the head are perceivable in visual information as “sweeping of the field of view (…) and the wheeling of the field,” but not, again, as felt motions, with which the visual experiences can enter into relations. As Heft writes, this means that “accompanying exteroception is always ego- or interoception,” or in Gibson’s own words: “The optical information to specify the self, including the head, body, arms and hands, accompanies the optical information to specify the environment. The two sources on information co-exist” (Gibson, 1979, p. 116). This, however, also refers to the body as an object; its movements, specified by the optical flow, are considered in terms of coupling to the processes in the world, but the body as experiencing, lived one, the proprioceptive or kinaesthetic information of felt body motion (which does not have to be specified by optical flow) does not seem to be a discernible element of experience and, for example, cannot be coupled to the experienced visual flow. This Gibsonian understanding of the body, in other words, is not equivalent to the felt, bodily presence that dominates the Husserlian notion of *lived experience*.

It can be argued that both ecological psychology and enactivism reject a mentalist version of agency and recognize the inseparability of agency from bodily activity. Yet, as we have shown, the body seems to be playing a different role in constituting experience for the two approaches, and this necessarily leads to distinct understandings of agency, to an analysis of which we turn in the next section.

## Experience, Embodiment, and Agency in Enactivism and in Ecological Psychology: Discussion

For both approaches, in the first instance, agency can be understood in the context of action and goal-directed movements. For phenomenologists, and enactivist too, our ordinary way of being in the world is primarily practical, which means that it is not only driven by practical concerns but is best described in those terms too (Gallagher and Zahavi, 2008, p. 153). The preoccupation with the “lifeworld,” i.e., with the daily, bodily, pre-theoretical world of experience, is there in Husserl, Heidegger, and Merleau-Ponty, and it aims to describe human experience as it is lived, that is, in terms of the actions and movements that having a body allows. But agency cannot be understood as linked exclusively with practical goals or given in intentional action. A phenomenological understanding of agency distinguishes between “an experiential sense of agency” and “an attribution of agency” that can be made when one is asked about one’s own actions (Gallagher and Zahavi, 2008, p. 160). The former is precisely a bodily-given sense of agency and is more basic than the latter, which depends on it. It combines a bodily given kinesthetic experience of movement and a sense of control of one’s own actions. The lived body is, according to Husserl, originally given in the awareness that I can move, although this awareness often remains implicit (Husserl, 1989, see also Taipale, 2014, p. 43). More specifically, Husserl (1989) describes our awareness of our own bodies as a field of sensing (*Empfindnisse*) whose role is to constitute our perceived body as our own. As commentators have noted, “Husserl rarely invented new terms, so this shows he was struggling to express something not captured in ordinary language. The term appears to bring together two other terms: *sensation* (*Empfindung*) and *lived experience* (Erlebnis)” (Moran and Cohen, 2012, p. 299). Sensings (*Empfindnisse*) are sensations in their immediate manifestation to the lived body (Husserl, 1989, Taipale, p. 44) but which also “communicate further some other object” (Moran and Cohen, p. 299). Thus, for example, seeing the blue of the sky is a way of “living-through” the experience of blueness and, at the same time, acknowledging the perceived object: the color of the sky as blue^7^.

This is best demonstrated on the example of the sense of touch. On the one hand, touch, as shown by David Katz, an experimental psychologist, uniquely among the senses utilizes agency, as when with our hands we produce the various tactile qualities that we experience (Katz, 1989, p. 242). Thus, Husserl maintains that active touch is critical for the very experience of having a body; the felt body (*Leib*) is “constituted originarily only in tactuality” (Husserl, 1989, p. 158). On the other hand, touch also allows for experiential duality: sensing is doubled in touch, as it allows the experience of touching and being touched in the same act of experience (Husserl, 1989, pp. 153, 155; see also Taipale, 2014, p. 48). Uniquely, in the sense of touch, Husserl claims, I can produce a sensation by moving and also localize it in my own body, i.e., I can touch myself touching in a way that I can never see myself seeing. This also makes possible the experience of the dual nature of the body – as both subject and object, a state of affairs that further allows us to understand sociality as a kind of bodily intersubjectivity (Husserl, 1989, p. 311). Kinaesthesia, on the other hand, does not constitute a separate field of sensing for Husserl because the sensation and the sensing cannot be separated. As Taipale (p. 29) says, “the kinaesthetically sensed is nothing other than the kinaesthetic sensing itself.”

As already noted, in phenomenology an implicit awareness of one’s body and of its motility constitutes a basic sense of agency, summarized in the Husserlian unproblematic “I can move” (Husserl, 1989). Before anything else the lived body is the expression of this original capacity for motility, which accompanies all feelings and sensations given within the somaesthetic field of experiences and is manifested in the non-purposive movements of stretching, breathing, yawning, or running. These are movements that can be passive or pre-intentional, or simply involuntary and reflexive, such as twitching, but are nevertheless always experienced as one’s own. As commentators have noted, with respect to what is meant by “kinaesthetic,” “Husserl is not referring to the physiological movements of the body (the physical range of movements of which the body is capable) but rather our first-person experiential sense of the moving of our eyes, tilting and turning the head, looking up or down and so on, especially in so far as these movements are *freely* undertaken” (Moran and Cohen, 2012, p. 181). When it comes to perceiving and acting in the world, this kinaesthetic experience, accompanying everything that one does, is not merely secondary to the given perceptual object. On the contrary, it is what makes possible the very constitution of the perceived world (Husserl, 1989). What is crucial in this context, as already discussed, is to acknowledge the fact that Husserl speaks of the mutual codependency existing between the world and the lived body that perceives it. In the words of Dan Zahavi, “we are aware of perceptual objects by being aware of our own body and how the two interact, that is, we cannot perceive physical objects without having an accompanying self-awareness, be it thematic or unthematic” (Zahavi, 2002, p. 18). So, while the reciprocity of subject/world relations is clearly evident here, as it is in ecological psychology, what seems emphasized in Husserl is precisely the bodily experience of the subject.

Husserl is preoccupied with the lived body as both an active doer involved in intentional acts and as a subject that is pre-reflectively or reflectively aware of itself. Depending on the type of movement it performs, the body is more or less present to awareness. Thus, everyday activities such as walking or eating are practiced without explicit awareness, and so are habitual actions or practical skills, such as writing or playing an instrument with fluency. These acquired capacities for movement are agentive but not to the same extent as when one is first learning to drive, for example. Thus, as other phenomenologists have also discussed, the body recedes in experience and attention moves toward the objects of perception. The agency of the body thus becomes experientially absent, or, as Husserl would call it, “passively active,” “an unthematized substratum from which the world is acted upon” (Leder, 1990, p. 19). But this *anonymity* or transparency of the body is not to be interpreted as the absence of agency, or the lack of bodily self-awareness. It is, rather, a different mode of agency and self-awareness, a *body schematic awareness* (Merleau-Ponty, 2002), or *operative intentionality*, a term used initially by Husserl but also adopted by Merleau-Ponty. The important point to be made here is that any form of agency is supported and ultimately made possible by a constantly present bodily-given experience, realized in the ability to move. In the context of this, we agree with Sheets-Johnstone’s (1999) assessment that Merleau-Ponty in his use of *operative* or *motor intentionality* neglects Husserl’s emphasis on the qualitative character of self-movement, i.e., on the quintessential role of “kinesthesis,” and prefers to think of movement as “a way of access” to the world (p. 243). According to Sheets-Johnstone, bodily intentionality, defined only in terms of a pragmatically given activity, cannot be a necessary or sufficient basis for embodied agency. We believe that enactivism does take this suggestion on board, when it comes to its proposals as to what constitutes agency.

It is fair to say that the question of agency represents a foundational and definitional concept for enactivism, both in the early work of Varela et al. (1991) and in subsequent developments (Thompson, 2005, 2007; Barandiaran et al., 2009; Buhrmann and Di Paolo, 2015; Stapleton and Froese, 2016). Agency, in the enactive approach, is defined under three main topics: *self-individuation (autonomy)*, *interactional asymmetry*, and *normativity*. As a feature of agency, *autonomy* points out to the fact that enactivism defines only living beings as cognitive systems^8^. As Thompson describes it, “living structures are ontologically emergent with respect to mere physical structures. They constitute a new order of nature that is qualitatively distinct from the merely physical order” (Thompson, 2007, p. 75). Living beings are thus “autonomous selves,” which are “not merely self-maintaining, like a candle-flame,” they are also self-producing, “including an active … boundary that demarcates inside from outside and actively regulates interaction with the environment” (p. 64). Such a formulation does not mean that an organism is understood as detached from its environment but that the interactions with the environment are seen to serve the purpose of the organism’s own self-individuation. Enactivist have borrowed a phrase from the philosopher Jonas (1966) to describe precisely the nature of that self-sustaining relationship with the environment: *needful freedom* (Di Paolo et al., 2010, p. 38). “Needful” explains the organism’s dependence on the environment for its sustainability, while “freedom” expresses its agentive autonomy in this very process. The enactive approach and phenomenology can be seen to converge on the issue of autonomy or self-individuation, despite the more encompassing level of description provided by enactivism. Autonomy is a fundamental characteristic of biological life, expressible through the capabilities of the lived body. Hence, agency in enactivism is clearly understood as being reliant on biological embodiment: “a genuine agent is biologically embodied” (Stapleton and Froese, 2016, p. 113), but it includes a conceptual shift toward a teleology of sense-making, of “enacting a world of significance and valence” (Thompson, 2007, p. 158) for an individual. Autonomy can be seen then as suggesting a strongly embodied sense of agency and an explicit sense of subjectivity, which puts its proponents firmly in the phenomenological camp. At the same time it allows for a discussion of biological, yet non-human, kinds of agency under the same umbrella, thus grounding human subjectivity in more basic forms of life and organic development^9^. Living organisms thus achieve autonomy through a precarious dependence on a world that is always experienced as value-laden for them. When the world is experienced as changed or deficient in some way, an organism can be seen to be in danger of losing its self-maintaining function, i.e., its autonomy, unless it adjusts its relationship with the environment in some way. It can be shown, therefore, that autonomy, thus understood, plays a crucial role in theoretical and practical work in, for example, psychopathology and bioethics. Treatment of patients as autonomous beings, and not as a set of symptoms, or considering psychiatric illnesses within their social and cultural contexts, are explicit attempts to think of patient autonomy in enactivist terms, i.e., in terms of a heterogeneously understood and phenomenologically grounded accounts of agency (for representative treatments, see Ratcliffe, 2008; Fuchs, 2010).

Another important distinction in the enactivist understanding of agency is *interactional asymmetry*. This notion describes the fact that organisms do things, they explore and perform with some regularity, and do not merely react to the world. “[F]or agency it is not sufficient for an individual system to just be a moving system, nor to merely be in interaction with the environment or other systems” (Stapleton and Froese, 2016, p. 118). It is the agent that drives the interaction, and it is the living, perceiving, and acting organism that at all times balances an openness to the environment with an agentive relation to it. The key issue here is that agency is seen as regulated by the agent and not as a passive response. Furthermore, the enactive theory of participatory sense-making shows that both this openness and this agency are intersubjectively achieved (De Jaegher and Di Paolo, 2007). Participatory sense-making thus explains how regulating one’s relation to the environment often is a matter of joint endeavor involving other agents. Sense-makers in interaction can then be seen to navigate two orders: that of their own agency and that of the interactive order itself (Popova and Cuffari, 2018). In this view, social interactions are seen as co-regulated processes between autonomous agents whereby relational dynamical patterns acquire their own autonomy (De Jaegher and Di Paolo, 2007, p. 493).

Finally, *normativity* in enactivism describes “the biological norms that guide adaptive behavior” (Stapleton and Froese, 2016, p. 119). This notion concerns the fact that organisms can either succeed in their dealings with the world or fail. Normativity thus expresses the need to acknowledge that there exists an optimal level of engagement between an organism and its world, one that the organism as an agent seeks but also, at times, fails to achieve. Normativity is fundamentally understood as a biological norm that guides adaptive behavior (Stapleton and Froese, 2016, p. 119) but can be seen to be operative in the form of values in not strictly biological terms, but, more specifically, in affective terms and at socio-cultural levels (see Colombetti, 2010). With this notion the enactivists come closest to a phenomenological sense of felt embodiment and to a Husserlian bodily subjectivity. Although often expressed in the language of adaptive dynamics, and norms geared toward some biological advantage, normativity should not be understood in strictly physiological terms but as a step toward a self-felt subjectivity.

It can be argued that with the notion of the interaction asymmetry enactivists can be seen to address the criticism voiced by Sheets-Johnstone toward Merleau-Ponty. An organism’s agency (both pragmatically and kinaesthetically available) is given a certain priority in relation to this organism’s own goals or norms and this is revealed in experience. As far as the authors of the present article could gather, this kind of experience of one’s own felt movement rarely features in the dynamical models of perception within ecological psychology, perhaps due to Gibson’s legacy described above.

We have already argued that an understanding of experience as “purely relational” in Jamesian radical empiricism gave Gibson’s ecological psychology the impetus to consider the relational aspect of organism/world engagement as primary. As Heft points out in his chapter on William James’s radical empiricism as a foundation for ecological psychology, “this analysis of the multiplicity of potential structures in pure experience, and of the selective function of knowing as the process by which some of these structures are realized, establishes the basis for James’s philosophy of radical empiricism as an alternative to metaphysical dualism” (Heft, 2001, p. 30). James’s understanding of the relation between the perceiver and the world embraced a kind of “phenomenal monism” (Edie, 1970), which, in turn, supports the pluralistic nature of “orders of reality.” This is a strong fundament on which the research program of ecological psychology is built.

Recognizing the richness of experience, its dependency on self-generated activity, and, most importantly, its relational character allowed ecological psychology to reach a deep and nuanced understanding of the embodied nature of cognition that mainstream cognitive science is still struggling to achieve. Moreover, the radical empiricist acceptance of direct perception of relations allowed for discoveries of complex relational variables that directly control our actions (Lee, 1976) and the unprecedented development of ecologically valid models of behaviors (see, e.g., Haken et al., 1985; Warren et al., 2001; Turvey and Carello, 2011), including behaviors in relation to brain activity (e.g., Jirsa et al., 1998; Kelso et al., 2013). The dynamical structures underlying action control are being uncovered, with due attention both to self-generated movement and to the affording character of the environment. Combined with dynamical systems tools for modeling behavior, this approach is presently advancing as one of the very promising scientific alternatives to the information processing approach in the cognitive sciences. Its strength lies both in a coherent theoretical background and in highly developed methods for dealing with complexity of the relational nature between an active body and the rich structures of the environment.

However, the same clearly expressed Jamesian provenance does not present itself readily when it comes to ecological psychology’s approach to felt experience as a basis for agency. As already noted, it seems that not all, often crucial, threads present in James’s account and later picked up by phenomenologists (see, e.g., Edie, 1970) are also present in Gibson’s work. For James, embodiment seems to be the origin of the multiplicity of experiences, including both the functionally oriented origin of physical activity, relating the changes in the environment to the experienced changes in the body and, at least with equal validity, the self-identifying “nucleus of our personal identity” (James, 1890, I, p. 341), “the fons et origo of all reality” (II, p. 296). The latter constitutes one of the aspects that was later developed in the philosophy of Husserl, Sartre, or Merleau-Ponty (Edie, 1970, p. 515). This richness does not seem to be fully reflected in the Gibsonian approach, where the emphasis on action in the world turned researchers’ attention to this outward function of bodily experience. Gibson recognizes the importance of proprioception (Gibson, 1966; Hamilton, 2013), but it is treated primarily as knowledge of body position and movement, to which the other senses can be related, and less as the reflection of a bodily-experienced selfhood.

These distinct ways the body is known will determine the way agency is understood. For James the origins of activity are in the experiences of the body: we experience the environment through our bodies and also experience our bodies through the environment (Heft, 2001, p. 55). The experience of motion provides the feeling of ownership present in all self-initiated action. It is this experience that, as Heft rightly notes, “was laying some groundwork for the significant philosophical analyses of later philosophers such as Merleau-Ponty” (Heft, 2001, pp. 55–56). This central self, the “self of selves,” as James called it, is to be found on empirical grounds and can be identified with the movements of the body (Heft, 2001, p. 56), envisaged as an intra-specific flow of bodily events, not with the body as perceived. This leads to experience being understood as consisting of the relation between two different dynamic dimensions that contribute to the ongoing stream of experience in James. One is the intra-specific flow of bodily events that we identify with the self, and the other is extra-specific flow of environmental features as we move through the world (Heft, 2001, pp. 56–57). Such a source of body knowledge is distinct from just knowing the optic flow caused by one’s own body movement (sweeping and wheeling of the field) or from specifying one’s own body as occluding edges (persistent features in the field of view), as we see later in Gibson.

Following James’s understanding of “pure experience” as neither subjective nor objective, it is possible to trace back Gibson’s definition of affordances to a congruent conception, expressing the implication of the perceiver in the very act of perceiving. To the extent that the lived body is seen as complicit in the act of perception and action in the world, the theory of affordances bears certain similarity to a phenomenological understanding of the body. But this is the phenomenology of action and behavior, of tool usage and pragmatic living, not the phenomenology of bodily selfhood, of pre-reflective embodiment and implicit, intransitive (passive) experience of bodily self-awareness. Agency in ecological psychology is thus understood in the context of this more pragmatic sense of embodiment, in relation to undertaken action.

In the context of our discussion of agency, it is helpful to mention Sheets-Johnstone’s critique of Gibson, specifically in relation to the notion of agency. She argues that “he transforms the phenomenon of movement into a phenomenon enmeshed in the global phenomenon of ‘perceptual affordances”’ (Sheets-Johnstone, 1999, p. 235). Movement (kinaesthesia) does not constitute a perceptual system for Gibson, the way the other five senses do, and therefore remains purely instrumental to them. For him movement is only the way we possess of “picking up information” that is there in the environment, not an experiential aspect of bodily existence (Gibson, 1979, p. 238, quoted in Sheets-Johnstone, 1999, p. 235). Ultimately, for Sheets-Johnstone, because Gibson chooses to focus exclusively on the five senses, and not on proprioception or kinaesthesia, he restricts his account of perception. Interestingly, Sheets-Johnstone sees the commonly observed focus on the environment that many commentators have pointed in Gibson’s work as stemming directly from his preoccupation with the senses, with “*what* we see, hear, smell, taste, and touch,” rather than with the living organism itself (Sheets-Johnstone, 1999, p. 236). Therefore he completely misses an opportunity to describe “the affordant kinetic power of the organism” as a system in its own right. The phenomenon of movement, that is, of self-movement, “as a phenomenon in its own right is elided” (p. 236). As she claims, “movement is something both more and other than instrumental, and … kinesthesis may afford something both more and other than information” (p. 238). Consequently, Thelen and Smith have later argued that “movement must itself be considered a perceptual system” (Thelen and Smith, 1994, p. 193). The kinesthetic experience is experience on its own right, at the core of constituting the agent (Sheets-Johnstone, 1999, p. 119).

To be sure, agency is one of the key notions for ecological psychology: grounding perception in self-controlled activity in the world has successfully changed the perspective not only in research on perception but in cognition in general. As Edward Reed notices, Eleanor Gibson lists agency as “the core phenomenon for psychology to explain” (Reed, 1996, p. 12), linking it to the key issues of autonomy and control. Yet the aspects of agency are analyzed here mainly as a capacity for action and defined in the context of actions’ properties: prospectivity, retrospectivity, and flexibility (Gibson, 1994; Reed, 1996; Turvey, 2019, p. 305). Even though the importance of spontaneous motion is recognized, especially for development of adaptive action (Turvey, 2019), it is discussed in terms of muscle activity, rather than felt motion. Edward Reed echoes Eleanor Gibson’s emphasis on the centrality of the notion of agency, which allows the organism to use opportunities for action rather than being fully determined by external causes: “The goal of ecological psychology is to *explain* the agency scientifically, not to explain it away, or to simply offer a discourse about it” (Reed, 1996, p. 19). But perhaps the stress on the “scientific” might have reduced the perceived explanatory potential of the felt, subjective, bodily experience, which rarely enters the theories of ecological psychology, despite Reed’s evident quest for the opposite (Reed, 1997). The question remains, however: Would involving bodily experiences, including kinesthetic self-perception, indeed make the explanation less scientific?

The aspects of agency important for the enactive approach described above, i.e., autonomy, interactional asymmetry, and normativity, would require recognizing self-felt experience, as they build not only on situated action but also crucially involve bodily experience of self and self-felt motion. Perhaps it is the lack of relating the perception-for-action in ecological psychology to the Jamesian stream of felt experience that diminishes the theoretical value of autonomy and asymmetry in the approach. Those aspects of agency that are set in inner experiential terms do not seem to be clearly defined. Agency of an organism, a basic premise for the ecological approach, on which natural selection and generic activity in the world are based, appears to be constituted more by environmental energy constraints than a drive, or a need, for self-maintenance. It can be argued that Varela et al. (1991, p. 203) were at least partly right when they criticized the ecological approach for not giving enough attention to how structural autonomy of an organism arises. It seems that only such structural autonomies can have experiences in the first place. Gibson explicitly said that he strives to build a psychology of values rather than a psychology of the stimulus (Hodges and Baron, 1992; Hodges, 2007) and understood values as ingrained in the perceptual fields of the organisms, indeed as directly perceived and guiding actions [see, e.g., fields of values in driving (Gibson and Crooks, 1938)]. It is difficult to see, however, how such experience of values could be based on picking up relations in the environment, even if such relations would include perceptions of one’s own movement when this movement is devoid of what Sheets-Johnstone calls the “ongoingness of primal kinetic liveliness” (p. 212).

It has been our aim in this article, through pointing out differences in conceptual understanding of the two frameworks, to argue not for a neat division of labor along existing lines, but for an integration of methodologies and mutual enrichment. From the discussion it should be evident that the ancestry of the two frameworks, respectively, the work of James and Husserl, had a lot of commonalities. Later developments, as shown in Gibson’s writings and the work of early enactivism, appeared to move away from each other, with different emphases and quite distinct research trajectories. Perhaps only now, with the later generations of both ecological psychologists and enactivists, we can observe not only a growing and far-reaching mutual interest, but an increased scope for compatibility, to which the current issue is a testament. Two good examples of the feasibility of this aim already exist, evidenced in the two steps toward each other made by an ecological psychologist and an enactivist, respectively.

The first is an attempt by a prominent ecological psychologist to capture felt experience and relate it to the origins of agency. Kelso (2002) laid the conceptual grounds and later developed a concrete dynamical model of activity in a baby-mobile paradigm setting (Kelso and Fuchs, 2016). The model is based on a phase-locking synchrony, relating self-generated and self-felt movement to the perceived movement of the objects in the environment. It is these dynamical couplings that become relevant or meaningful and not just the relational variables in the environment *per se*. The systemic landscape for such couplings is usually multi-stable, with several possible coordinations, which can be selected according to tasks or other factors. The model is briefly described below in our analysis of agency in social coordination.

On the enactivist side, Ezequiel di Paolo and colleagues extend the work on sensorimotor contingencies (e.g., Nöe, 2004) to account for agency (Buhrmann and Di Paolo, 2015). The account is closely related to ecological psychology’s non-representational approach to perception as based on “skillful use of the regularities governing active exploration of the world.” Experience of oneself as an agent “derives from the ways in which we establish, lose, and re-establish meaningful interactions between ourselves and our environment.” This depends on a skillful control of the sensorimotor contingencies. Recognizing this relational nature of agency opens the way to ecological-psychology-like analyses of the structure of the environment in relation to structures of experience, with the self-felt bodily experience as an indispensable part of it. The dynamical systems tools, used and developed within ecological psychology to deal with complexities of multi-relational structures in terms of their global-local, multi-stable dynamics, are acknowledged as especially useful by the authors. This unlocks the way to identifying the dimensions of the contingencies, which themselves might be relational and non-obvious, thus opening the approach to the richness of structure present in the environment and within the organism/environment system.

In the next two sections we present two examples of domains in which we can observe the relative usefulness and potential integration of theoretical and methodological resources from the two approaches.

## Social Affordances and Social Agency in Early Interactions

Neither enactivism nor ecological psychology at their beginnings took account of the social reality that humans are constituted within. For ecological psychology the interest concerned mainly the social nature of artifacts (see, e.g., Costall, 1995) and socially constructed environments (Reed, 1995, 1996), with a stronger acknowledgment of the active, engaging nature of the social realm in which actions of humans take place in the work of Hodges and Baron (1992), where they flesh out Gibson’s understanding of affordances in terms of values. Perceiving and acting upon affordances was considered a value-realizing activity, which was illustrated in situations of social interaction. The abovementioned work by Heft (1989), which linked the notion of affordances to intentionality, opened interesting avenues for accounting for social interaction. In the empirical work within ecological psychology, social factors were considered in how social situations change affordances (Marsh et al., 2009), and how other people’s behaviors can be treated as opportunities for interaction (Valenti and Gold, 1991; Rączaszek-Leonardi et al., 2013). In enactivism, the crucial value of the social came with the notion of participatory sense making (De Jaegher and Di Paolo, 2007), although social concerns have always been a prominent part of phenomenology, commencing with the notion of *intersubjectivity* in Husserl.

The importance of the social realm is perhaps most evident while researching developmental processes. From an ecological perspective, developmental processes are understood as the tuning of the infants’ perception to the important information in the environment, which is dependent on the activity of an infant. One quickly realizes that the infants’ surroundings consist predominantly of other people in interaction with a child, constituting a kind of reliable and highly structured “social physics” (Rączaszek-Leonardi et al., 2018). Unlike the physical world, though, the actions of others most often engage the infant as a vital actor within the events.

Drawing on ecological psychology and especially its later developments, mentioned above, which linked perception of affordances to intentional action, researchers have shown how a social agent develops, tuning to the perception of social affordances (Rączaszek-Leonardi et al., 2013). It has been shown how the sensitivities of the child can be shaped, in a sense, “movement first.” On the one hand, almost every action performed with a child is an enaction of an interactive event, in which an infant is given a particular role that has to be filled with a particular action (e.g., smile) at a particular place of a sequence (e.g., after a gaze at an infant and calling her name), and with particular timing (otherwise repetitions and repairs follow). This way, infants learn that the particular movements of others are affordances for their own actions. On the other hand, infants’ spontaneous actions can become affordances, in the sense of being parts of an intentional act, also “movement first,” without developing “theories of mind” or other complex representational schemas. This happens when a random movement of a child is picked up by a caregiver and enveloped as an element of a sensible interactive event. Reliable enaction of such causal structures around infants makes them agents, who, with time, realize that their own movements afford actions for others. This is a story told within the ecological approach, showing how others’ movements become affordances for a baby and how baby’s own movements become affordances for others. It shows the dependency of perception on action and the immersion of action in socially reenacted intentional episodes, which give them meaning. Yet it seems that something is missing in the transition from merely perceiving contingencies of infant’s own movements with those of others to becoming an agent, realizing the affordances-creating potential of one’s own behavior.

It seems crucial for the development of agency in such situations that it is shaped not only by immersing an infant in structured interactive episodes, but that this structure is related to the felt experience of the moving body. Those are infant’s own movements, not abstract behaviors, that are embedded, enveloped by enactments of con-specifics, and thus this felt experience of the body can enter in relation with the perceived movements of the infant’s body and of the movements of others. The caregivers respond to the spontaneous movements of the baby with “the other part of the story,” or they demand a particular activity, co-creating sensible episodes with the child, which then leads to educating perception in purposeful intersubjectivity, but the foundational kinesthetic perceptual consciousness never ceases to underlie them. It should be mentioned that such education of movement and perception is not only instrumental: this would be underappreciating the kinds of constraints that are passed in the infant-caregiver enactions. The structures of events are such that the joint dynamics of acting bodies become not only functional or efficient but, above all, preserving crucial values for co-existence and co-action, such as mutual respect and relative agency in a situation (Rączaszek-Leonardi and Nomikou, 2015). These seem especially strongly guarded by the felt bodily experiences, the kinesthetic feelings in relation to unfolding events, and these, in turn, can be experienced as feelings of connection and joy, disconnection and despair, surprise, awkwardness, adequacy. These are felt experiences that can well become crucial parameters, e.g., in explaining the timings and intensities of joint enactions and, therefore, in guiding agents’ behaviors.

As already mentioned above, this kind of experience of one’s own felt movement is usually absent from dynamical models of perception within ecological psychology. Recent attempts, however, seem to capture them within a precise mathematical model of empirically studied phenomena (Kelso and Fuchs, 2016). In the model, agency is seen as emerging from spontaneous activity, movement first, relating in self-organized couplings both the internal feelings of one’s own bodily movements, the perceived self-movements, and contingent movements in the environment. In this complex relation “we discover ourselves in movement” (Kelso, 2002, after Sheets-Johnstone), see ourselves as agents, capable of effectuating the changes. “[T]he sense of self emerges as an explicitly *collective effect*” (Kelso and Fuchs, 2016, p. 51), spanning the infant and the movement of objects in the environment. Coordinative dynamics drives patterns of coordination, following the general pattern-formation principles in nature. This can be modeled by a system of differential equations relating in a bidirectional, informational way the oscillatory movement of the infant to the oscillatory movement of, in this case, a baby mobile. The thing that seems crucial and that opens the possibility for the felt motion to impinge on the resulting dynamics is that the coupling between the two oscillatory movements depends on a parameter, which seems to relate the mobile salient motion to both kinesthetic information from leg movement and haptic information from the baby’s body. Kelso calls the parameter attentional (“baby’s attention to self-generated movements and the kinesthetic, visual and auditory consequences they produce,” p. 51) but it is crucial that the attention relates what is the sensed experience of the moving body to the body as perceived (haptic information), and to the movement in the environment. A critical value of this parameter leads to the “eureka” effect, a discovery that it is “I” who makes the mobile move. What is shown in other experiments is that the sense of one’s own body movement is crucial for the couplings to emerge, while the haptic information is less so. The coordination of the felt, experienced, movements of the baby and the movements of the mobile that are in a co-regulating positive-feedback loop leads to the emergence of agency, in which the baby, within several minutes, discovers the effects of the kicks and triples their frequency with visible delight.

Returning to the early interaction situation, the picture of learning to perceive and act upon social affordances “movement first” is enriched by noting that these are one’s own felt movements that are met with the enactions of caregivers. This feeling of movement becomes enfolded in an enacted project. In social situations we thus learn not only how our body should move in a given point of a social event, we learn how our body should feel in such movements. As Sheets-Johnstone has argued, movement comes before behavior: a behavior (instrumental) is “a kinetic episode that we, as adults, partition off from the global phenomenon of animation” (1999, p. 212), while what is constitutive to our conscious self is the “ongoingness of primal kinetic liveliness,” which leads to a “foundational kinesthetic perceptual consciousness.”

Shaping the infant’s perception for social interaction thus in a sense relies on the baby having particular bodily kinesthetic experiences, and makes them social through relating them to enacted events. This felt kinaesthesia in enaction with others provides for the emergence of the baby as a social agent: the infant not only feels that she moves and how this feels but also that she becomes a mover, also in connection to the inner feelings. Lived bodily experience thus gives access to the direct feeling of the valence of a particular engagement with the world, which one’s own perceptions of external events only does not provide.

## Affordances, Agency, Art

There is an area of profound and significant human experience that lies beyond the perception/action cycles, as described in ecological psychology. This is the area of art production and reception. In the brief space we have here we will only suggest some ways in which enactivism can be seen as being able to enrich ecological psychology in the study of this irreplaceable aspect of human life. When speaking about art, we will not be providing a definition of art, a consensus on which is still forthcoming after centuries of discussions, and talk instead of “artful practices” that include a broad spectrum of activities, such as dance, theater, painting, sculpture, video installations, and the like. In a similar vein, the study of aesthetics has grappled with the question of what is the essence of all things we call “beautiful,” without much agreement, and this discussion will not detain us here. It is sufficient to mention that Alexander Baumgarten introduced the term *aesthetics* as early as 1735, and defined it to mean “a science of how things are to be known by means of the senses” (*scientiam sensitive quid cognoscendi*) (quoted in Guyter, 2004, p. 15). Aesthetics is then a scientific study of sense perception in relation to the fine arts and other objects of beauty, and, importantly, it provides knowledge that is understood to be both thought and felt. Another way to say this is to acknowledge that aesthetic reception is undoubtedly “a refined and intensified form of experience,” as Dewey (1934) has claimed. Indeed, aesthetic reception is about perceiving the world and its objects, but it is nevertheless a particular kind of perceptual process. We have argued that ecological psychology and Gibson’s notion of *affordances* describes perception in terms of action and everyday engagements with the environment. Yet, practical interests in an object do not exhaust our ways of engagement with it. Art objects, whether they are pictures, sculptures, installations, or dance performances, are unique. While the act of looking at a painting, for example, with its specific processes of moving the eyes, fixating and focusing them, and the repetition of these processes, might be compared with how we look at an object in the world, the aesthetic artifact remains somehow an autonomous and alien entity, removed from the ordinary world, and able to produce a distinct experience that nevertheless brings us back to the world. For Dewey works of art are not objects or events designed for observation but, rather, “the actual work of art is what the [art] product does for experience” (Dewey, 1934, p. 9). In both making and engaging with art works, Dewey says, “we are carried out beyond ourselves to find ourselves” (p. 199).

The uniqueness of the art object or art practice can be described in at least two ways. First, in the terminology of Russian formalism, they contribute to “defamiliarization” of everyday experience: they work against habituation to uncover forgotten, sensory aspects of “being in the world.” As Victor Shklovsky, one of the main theoreticians in this area, has put it: “[t]he technique of art is to make objects ‘unfamiliar’, to make forms difficult, to increase the difficulty and length of perception because the process of perception is an aesthetic end in itself and must be prolonged” (Schkovsky, 1917, p. 12). The operative word here is “ostranenie,” or “making strange” precisely that which appears ordinary in experience. Second, as described by Bence Nanay, a philosopher of perception, art objects require a distributed form of attention: because the aesthetic object and event is removed from everyday experience (i.e., is perceived as framed differently), we are both focused on the object and paying attention to everything that this object is or, potentially, can be (Nanay, 2016, pp. 24–25). Importantly, in both these descriptions, subjective experience underlies the process of aesthetic reception. On the side of production, art is commonly associated with strong expressions of subjectivity. As a receiver of art one also experiences a strong sense of being an agent who feels the “strangeness” of the given art form at the same time as trying to make sense of it.

The concept of affordances has been used on a few occasions to describe human engagement with architectural design and even art objects. For example, Withagen et al. (2012) argue against the view that “affordances are mere action possibilities” and propose instead that affordances are understood as potential invitations for actions, providing examples from industrial design and architecture. Baron et al. (2008) offers a new term, *tentativeness*, which describes a shift toward a more active and participatory way of engaging with particular examples of visual art, sculpture, and architecture. This is a solid argument against automaticity in perception and for a more bodily response to art that is by its very nature inviting not just visual but also kinesthetic reception, such as Serra’s sculptures and the architecture of Arakawa and Gin. Kadar and Effken (2008) use paintings by Cézanne and Hokusai to show how active participatory perception can be enhanced by the use of particular drawing techniques. They argue that classic linear perspective relies on static and passive perception on the part of the viewer, while Chinese (parallel) perspective mirrors somewhat the dynamics inherent in visual perception. Similarly, they argue that Cézanne uses visual distortion in both still lives and landscapes, thus aiming to enhance the affordance structure of the painted objects. These are valuable contributions linking visual perceptual knowledge with certain aesthetic values, and with questions about how sense-making of pictorial artifacts happens. The suggestion that the point of view in the visual arts can be understood as an affordance for the viewer is not, however, unique to ecological psychology and has been proposed as a comprehensive relation between the viewer and particular developments in the history of Western visual art by, for example, art psychologist Ciaran Benson. Thus, a medieval artwork presents a flat pictorial space that does not require a particular perspective, while the geometry of linear perspective used in the Renaissance requires a particular “entry point” into the work (see Benson, 2001). This particular point of view becomes significant and often carries a symbolic meaning expressed by a particular pictorial arrangement and mirrored by the viewer’s eye. The question of the viewer’s point of view turns out to be a lot more complicated when we get to modern and experimental art, where no particular way of looking is required by the spectator, and viewing art becomes, rather, a matter of personal choice of a way of engagement. This is where the subjective experience of perception, with no specific, pre-defined point of entry or even way of interaction with the artwork, takes the lead. A case in point is art where subjective visual perception itself (how I experience myself seeing) becomes the object of artistic presentation (as in the experimental work of James Turrell), as well as other kinds of art, like sculpture, physical theater, or improvisational dance, where self-felt embodiment necessarily accompanies reception.

To that extent, attempts by ecological psychologists to highlight the active and participatory perception of art objects are at best partial explanations of why certain visual properties (e.g., perception of point of view in a painting, or patterns of activity in moving through a building) constitute a part of aesthetic reception. Heft has described the relationship between an affordance and behavior as that of “fittedness and compatibility”: while affordances do not elicit behavior, they can still prompt an act (Heft, 1989, p. 10). The question that arises here is one of an alternative scenario, namely, when such “fittedness and compatibility” do not happen readily, or provide a multiplicity of options to be taken. When, in other words, an observer is faced with an object or event that they cannot comprehend immediately, after a prolonged exploration, or not at all. So, while applicable to certain aspects of visual art and architectural design, using the terminology of affordances in relation to art remains so far only an incomplete account of how we make sense of it, as it does not touch on topics such as self-movement, affectivity, or intersubjectivity in processes of art creation and reception.

With their emphasis on human experientiality and the complexity of human agency involved in art practices, enactive theories are better placed to explain not just how we perceive art but also how we experience it. There have been attempts to provide enactive accounts of reading a fictional narrative, albeit in quite distinct ways (Caracciolo, 2014; Popova, 2015; Popova and Cuffari, 2018), reading poetry (Popova, 2016), the movement-based pedagogy of Jacques Lecoq (Murphy, 2019), and of human communication broadly conceived (Di Paolo et al., 2017), to mention just some examples. A particular feature of the enactive approach to cultural forms is the heterogeneous notion of agency that is taken into account. In engaging with art, the individual agency of the viewer, listener, or participant is balanced by an autonomous dynamic interaction with the art object that arises in the very exchange with it. As discussed, a characteristic of art works and practices is both the experience they initiate and the non-instrumental nature of the engagement they provoke, discernible in a constantly modulating sense of “being in control,” of knowing and making sense of the particular encounter. Such engagement is normative and asymmetric for the viewer, yet, making sense of an art object invariably has a participatory character, which involves a distributed attentional effort (agency on the part of the experiencer) and lies outside of immediately situated space, time, and instrumentality, i.e., is detached in some way. It is also constituted intersubjectively: in art we engage not just with the object but in some way also with the creative agency of the artist, embodied, so to speak, in that particular artifact. Di Paolo (2016) has argued persuasively for “participatory object perception,” where even immediate instrumental use should be seen as secondary to a dynamic of social practices, involving those objects. As Di Paolo has described it with respect to object perception generally, but, as we see it, with potential wider applicability to art, “it is a social skill that I enact individually” (Di Paolo, 2016, p. 253). The intersubjective aspect of any form of art, given in the dual constitution of materiality (of the body, the canvas, the stone), shaped by the agency of another, and in concretization brought about by the agency of a perceiver (or participant) is particularly well-suited to enactivist treatment. The valuable lessons from ecological psychology about, for example, the spatial organization of pictorial space and active perception can only be enhanced by considerations of affectivity and experientiality that enactivism can bring to the table in discussions about art.

## Conclusion

What can be learnt from such comparison, as we have offered, between the two frameworks under consideration and where to go from here? Perhaps it can be claimed that James’s radical empiricism played for Gibson’s ecological psychology the role that a phenomenological understanding of the body played for enactivism. Thus, for Gibson radical empiricism enabled direct perception and took away the necessity of information processing by allowing relations to be directly apprehended. For enactivists, a phenomenological understanding of the body led to a heterogeneous notion of agency that includes a felt sense of movement and bodily action, yet is consistent with a broader subjectivity linked to a defined perspective and self-generated normativity.

In the two approaches, ecological psychology differs from enactivism in how it understands cognition to be linked to the body (i.e., is embodied), namely, through a functional link to activity in a complex, structured world, rather than to the felt, qualitative self, discovered in movement. This is visible in how Gibson, for example, describes perception of self, or ego- or intero-ception, not as proprioception but as perception of one’s nose, arms, hands, torso, etc. It is an important perception for establishing relational variables, including the movement of the subject, but it is not the same as the felt subjectivity of movement. On the other hand, enactivism differs from ecological psychology (or at least seems to be less specific about it) about what embodiment includes. As we have shown, ecological psychology carefully specifies the relational nature of the world, the rich structure, which can be directly picked up by the organism. The key point of the relational nature of the Jamesian structured and still directly experienced environment might not be capitalized on in phenomenological and thus enactive thought, and the non-obviousness (for experience and for description) of the complex relations that govern action in the lived world might thus be underappreciated. An important aspect of this might be the lack of sufficient concern for the relations the body itself enters in perception, i.e., the scope of how the body is implicated in the much criticized “information pick-up” from the world that enactivists generally describe ecological psychology to be about.

It seems that this rich relationality and careful consideration of the environmental structure could benefit immensely from including, as a backbone for all other relations, the stream of human presence, with its directly felt quality. On the part of ecological psychology this would require admitting that direct perception is also a direct access to felt kinesthesia, and the relations it enters into with both perception of one’s own movement and the movements of the world. A heterogeneous notion of agency, such as has been developed in enactivism, with the notions of autonomy and asymmetry, might be beneficial for recognizing different kinds of engagements within the environment in ecological psychology. Yet, the importance of agency has to be complemented by attention to how this environment is richly structured and ubiquitously present.

For joining the efforts of the two respective fields, more is needed than an acknowledgment of an existing apparent division of labor, which would seem natural given the respective histories of the fields (Baggs and Chemero, 2018), namely, that ecological psychology takes on the identification of the complex and relational structure of the environment, and specification of its informational value and coupling to the organism, while enactivism develops an increasingly elaborate study of kinds of human experiences and embodiment. This apparent division is not a result of “a major philosophical barrier to unification” (Baggs and Chemero, 2018), as the above discussion of the radical empiricism of William James clearly demonstrates, but stems from specific understandings of the role of embodiment in experience. Rather than continue a divide along established lines and accept specializations, what is needed, we claim, is a careful analysis that will make explicit how the two frameworks approach the relation between the contents of experience, especially experience of one’s embodied self, and the kinds of embodiment and agency that an organism commands. Thus, instead of continuing with their own tasks to solve, each approach requires a push for a better awareness of their conceptual core and, if necessary, for a change in concept definitions and terminology. The key to integration, we believe, is a reconciliation of the importance of felt experience with the structured ecological information available in the environment. Despite some complementarity of efforts already in evidence, the project of integration remains a strong challenge for the future.

## Author Contributions

YP and JR-L contributed equally to parts introduction and conclusion. YP wrote parts enactivism and affordances, agency, art of the manuscript. JR-L wrote parts ecological psychology and social affordances and social agency in early interactions. YP wrote a substantial part of experience, embodiment, and agency in enactivism and in ecological psychology: discussion. Both authors contributed to the article and approved the submitted version.

## Conflict of Interest

The authors declare that the research was conducted in the absence of any commercial or financial relationships that could be construed as a potential conflict of interest.

## Footnotes

1 The term *intentionality*, as a distinctive feature of consciousness, stems from the Latin verb *intendo* and means “to aim” or “to stretch” and therefore describes how consciousness can be directed to objects that are internal, such as memories or anticipations, and also external, such as things and events in the world. Husserl borrowed the term from his teacher Franz Brentano, although he considered Brentano’s understanding of it misleading. Its use in phenomenology indicates the necessity to bridge a dichotomy between lived experience and the way it always points beyond itself.

2 It needs to be noted that the branch of enactivism that we are discussing is the one developed from the original work by Varela et al. (1991, 2016), and evidenced in subsequent work by Thompson (2007); Di Paolo et al. (2017), De Jaegher and Di Paolo (2007), and Buhrmann and Di Paolo (2015), among others. “Sensorimotor enactivism,” as it is sometimes termed, is a variety of enactivism that concentrates on the study of environmental interactivity and sensorimotor contingencies and does not undertake to explore issues of subjectivity to the same degree. Representative theorists in the latter include Hutto and Myin (2012); Nöe (2004), and O’Regan and Nöe (2001). For a description of varieties of enactivism, see Ward et al. (2017).

3 It is important in a paper concerned with influences and historical connections of ideas to emphasize that it was Husserl’s own phenomenology that can be considered a first true phenomenology of embodiment. As Moran (2017, p. 28) observes, this fact is still not appreciated fully by researchers, with some notable exceptions (e.g., Taipale, 2014, the work of Dan Zahavi), due to the fact that Husserl did not publish the work contained in *Ideas II* (1989) in his lifetime. As a result, Merleau-Ponty, who had access to Husserl’s manuscript as early as 1939, is usually credited with initiating the phenomenology of embodiment in his *Phenomenology of Perception.*

4 An example of such an attempt is Varela’s project of neurophenomenology (also known as the project of *naturalization in phenomenology*), which aims to combine the experimental subject’s 1st-person account of her experience with neuroimaging data. See Varela (1996) and Varela and Sheer (1999) for descriptions of the whole research project.

5 The relation of Gibson’s work to phenomenology reflects well this complexity, which is one of the main points in this paper article. He might have been influenced by Merleau-Ponty on a general level, and it is true that the general assumptions of his theory, especially about the relation organism/environment, can be seen to parallel the work of Merleau-Ponty (Heft, 2001; Chemero and Käufer, 2016). On the other hand, as we will claim below, some threads present in James’s work, congruent with the phenomenological approach, seemed to not have been picked up by Gibson.

6 Note that we use “functional” here in its psychological sense, as it applies to the psychological approach of James (1890); Dewey (1934), and others. This is distinct from “philosophical functionalism” as used in contemporary cognitive science. See also note 8, below.

7 It is thus very important here to differentiate a phenomenological, and particularly Husserlian, understanding of “sensation” from a more traditional and widely used in psychology notion. For Husserl sensation constitutes the incarnate relation of the self with the world, while for psychology, especially as criticized by ecological psychology researchers, it is a momentary, and “anatomically specific product of sensory receptor stimulation” (Heft, this issue, see also Turvey, 2019). Enactivism, as we show below, follows the Husserlian tradition in its insistence on the necessity of both a dynamic sensorimotor account of perception and a phenomenological account of bodily consciousness (Thompson, 2007). There are important differences in how sensations are treated in enactivism and ecological psychology, respectively, and our article focuses on one element of this issue, namely, the role of felt bodily experience in agency. As raised by one reviewer, the broader issue of sensations deserves further analysis, and the article by Harry Heft (this issue) is a very good starting point for such a debate.

8 From what has been said so far it should be evident that enactivism in the form discussed here is not to be equated with existing formulations by philosophers and cognitive scientists of what has been termed “extended cognition” (Clark, 1997; Clark and Chalmers, 1998). This position is most simply described as the belief that the environment and its resources can, under certain conditions, be considered as legitimate constituents of cognitive processes. In this view, cognitive processes are understood as functional and can be realized on human brains or any other “thinking” devices. As Chemero and Käufer (2016) note, “the hypothesis of extended cognition is a natural corollary of philosophical functionalism” (p. 56). It needs to be pointed out, however, that with its emphasis on experience, enactivism is not a theory that can embrace philosophical functionalism in any form. The co-constitution of body and world, as defined by Di Paolo, Thompson, and other enactivists, does not entail that objects outside the biological body are parts of the cognizing organism. Thus, the relational character of the cognitive processes is not taken to presuppose any kind of ontological claim about the boundaries of the mind.

9 As some theorists have claimed, some of the foundations of the enactive theory of agency can be traced to Maturana and Varela’s (1987) work on the biological roots of cognition and particularly to the concept of *autopoiesis.* The latter literally means “self-creation,” and describes a form of organization of the living system, the most minimal of which is the living cell, that is both self-sustaining and self-generating. It has led, however, to some disagreements regarding the equivalence of autopoietic, living, and cognitive systems (Thompson and Stapleton, 2009; Froese and Di Paolo, 2011), which so far remain unresolved.
